# Treatment with decitabine induces the expression of stemness markers, PD-L1 and NY-ESO-1 in colorectal cancer: potential for combined chemoimmunotherapy

**DOI:** 10.1186/s12967-023-04073-y

**Published:** 2023-03-31

**Authors:** Nassiba Taib, Maysaloun Merhi, Varghese Inchakalody, Sarra Mestiri, Shereena Hydrose, Karama Makni-Maalej, Afsheen Raza, Fairooz Sahir, Fouad Azizi, Parveen B. Nizamuddin, Queenie Fernandes, Zeenath Safira K. M. Yoosuf, Salam Almoghrabi, Lobna Al-Zaidan, Alaaeldin Shablak, Shahab Uddin, Cristina Maccalli, Mohammed Ussama Al Homsi, Said Dermime

**Affiliations:** 1 Translational Cancer Research Facility, National Center for Cancer Care and Research/Translational Research Institute, Hamad Medical Corporation, 2030 Doha, Qatar; 2 National Center for Cancer Care and Research, Hamad Medical Corporation, 2030 Doha, Qatar; 3 Translational Research Institute, Academic Health System, Hamad Medical Corporation, 2030 Doha, Qatar; 4 Translational Research Institute and Dermatology Institute, Academic Health System, Hamad Medical Corporation, 2030 Doha, Qatar; 5 Laboratory Animal Research Center, Qatar University, 2713 Doha, Qatar; 6 College of Medicine, Qatar University, 2713 Doha, Qatar; 7 Laboratory of Immune and Biological Therapy, Human Immunology Department, Research Branch, Sidra Medicine, 26999 Doha, Qatar; 8 College of Health and Life Sciences, Hamad Bin Khalifa University, 34110 Doha, Qatar

**Keywords:** Colorectal cancer, Decitabine, NY-ESO-1, PD-L1, Stemness markers, Chemoresistance, Immune escape

## Abstract

**Background:**

The mechanism of tumor immune escape and progression in colorectal cancer (CRC) is widely investigated *in-vitro* to help understand and identify agents that might play a crucial role in response to treatment and improve the overall survival of CRC patients. Several mechanisms of immune escape and tumor progression, including expression of stemness markers, inactivation of immunoregulatory genes by methylation, and epigenetic silencing, have been reported in CRC, indicating the potential of demethylating agents as anti-cancer drugs. Of these, a chemotherapeutic demethylating agent, Decitabine (DAC), has been reported to induce a dual effect on both DNA demethylation and histone changes leading to an increased expression of target biomarkers, thus making it an attractive anti-tumorigenic drug.

**Methods:**

We compared the effect of DAC in primary 1076 Col and metastatic 1872 Col cell lines isolated and generated from patients’ tumor tissues. Both cell lines were treated with DAC, and the expression of the NY-ESO-1 cancer-testis antigen, the PD-L1 immunoinhibitory marker, and the CD44, Nanog, KLF-4, CD133, MSI-1 stemness markers were analyzed using different molecular and immunological assays.

**Results:**

DAC treatment significantly upregulated stemness markers in both primary 1076 Col and meta-static 1872 Col cell lines, although a lower effect occurred on the latter: CD44 (7.85 fold; ***p = 0.0001 vs. (4.19 fold; *p = 0.0120), Nanog (4.1 fold; ***p < 0.0001 vs.1.69 fold; ***p = 0.0008), KLF-4 (4.33 fold; ***p < 0.0001 vs.2.48 fold; ***p = 0.0005), CD133 (16.77 fold; ***p = 0.0003 vs.6.36 fold; *p = 0.0166), and MSI-1 (2.33 fold; ***p = 0.0003 vs.2.3 fold; ***p = 0.0004), respectively. Interestingly, in the metastatic 1872 Col cells treated with DAC, the expression of both PD-L1 and NY-ESO-1 was increased tenfold (*p = 0.0128) and fivefold (***p < 0.0001), respectively.

**Conclusions:**

We conclude that the upregulation of both stemness and immune checkpoint markers by DAC treatment on CRC cells might represent a mechanism of immune evasion. In addition, induction of NY-ESO-1 may represent an immuno-therapeutic option in metastatic CRC patients. Finally, the combination of DAC and anti-PD-1/anti-PD-L1 antibodies treatment should represent a potential therapeutic intervention for this group of patients.

**Supplementary Information:**

The online version contains supplementary material available at 10.1186/s12967-023-04073-y.

## Background

Despite the advances in diagnosis and treatment strategies, colorectal cancer (CRC) remains the second leading cause of cancer-related deaths worldwide (9.4%) and ranks third (10%) in terms of newly diagnosed cases [1, 2]. Currently, chemotherapies mainly comprising 5-fluorouracil (5-FU), irinotecan (IRI), and oxaliplatin (OX) represent the standard of care for the treatment of CRC patients [3]. However, most patients show chemoresistance, resulting in disease progression with a 5-year survival of less than 10% [4].

Several studies have suggested that a subpopulation of cancer cells, called cancer stem cells (CSCs), is responsible for chemotherapy resistance in CRC [5–8]. CSCs possess unique characteristics, including self-renewal, infinite proliferation, and multi-lineage differentiation capacities [9]. These features are crucial in cancer initiation, conventional therapy resistance, post-treatment-recurrence, and metastasis development [10]. Therefore, it is important to identify approaches in combination with conventional therapy for eradicating cancer cells and overcoming CSCs resistance [11]. Colorectal CSCs can be identified via cell surface markers such as CD44, CD133, CD166, Lgr5, ALDH1, and EpCAM [12, 13]. Other more universal CSCs markers including Nanog, Sox2, Oct-4, CD51, CD24, CD26, and CD29 were also reported [13]. Importantly, recent studies demonstrated that the expression level of CSCs markers such as CD44, CD133, CD166, Nanog, Oct-4, and ALDH1 are promising prognostic markers that can predict the clinicopathological features in CRC patients [14–19].

Immunotherapy using immune checkpoint inhibitors (ICI), including the programmed cell death protein 1 (PD-1), and its ligand programmed cell death ligand (PD-L1), has shown promising results in many different malignancies such as melanoma and lung cancer [20–22]. However, the response rate to ICI treatment in CRC patients is limited and is approved mainly for those with mismatch repair deficiency (dMMR) and high mutational burden [23–25]. The mutations can lead to the expression of neoantigens by tumor cells, which can then be recognized and targeted by the immune system leading to a robust anti-tumor response [23–25]. Similar to these neoantigens, cancer-testis antigens (CTAs) expressed by cancer cells (but not by normal cells) have been shown to represent high immunogenic antigens and are efficient targets for anti-tumor immune responses [26]. Among these CTAs, the New York Esophageal Squamous Cell Carcinoma 1 (NY-ESO-1) antigen has been reported to be highly immunogenic since it induces both humoral and cellular immune responses [27]. Therefore, NY-ESO-1 is considered a crucial immunotherapeutic candidate for cancer therapy. In CRC, the NY-ESO-1 mRNA and antibody expression have been documented to be suboptimal (9.9% and 24.5%, respectively) [28, 29]. Since NY-ESO-1 is an essential immunotherapeutic target, various strategies to circumvent its poor expression in CRC have been investigated.

Several drugs are being actively investigated for their role in inducing pathways that enhance the expression of target antigens which can elicit anti-tumor immune responses against CRC cells. Of these, chemotherapeutic demethylating/hypomethylating agents are of particular importance as they can induce changes at the genetic level leading to an enhancement of the anti-tumor response in cancers [30]. Decitabine (DAC), or 5-aza-2’-deoxycytidine, is a demethylating/ hypomethylating deoxycytidine agent used as an anti-cancer drug [30]. DAC covalently binds to methyltransferases and traps enzymes in the DNA, thus acting as an irreversible inhibitor of their enzymatic activity [31]. This effect leads to marked DNA hypomethylation and a reversal of silenced histone code at the tumor-suppressor gene loci (CpG islands). These dual hypomethylation–histone changes would also lead to significant upregulation of genes not silenced by CpG island methylation. Thus, DAC affects the expression of genes associated with silencing promoter-associated methylation and targets genes through its silencing-independent activity [32]. Furthermore, DAC exhibits a favorable toxicity profile, thus making it an attractive anti-cancer drug [32]. Currently, DAC is utilized for the treatment of several hematological malignancies, such as myelodysplastic syndrome (MDS) and acute myelogenous leukemia (AML) [33–36]. However, its therapeutic potential in solid tumors is still under investigation [37].

Long-term exposure to anti-cancer drugs creates a multidrug-resistance tumor that limits chemotherapy's effectiveness. It is reported that long-term exposure of HCT116 cells to DAC confers resistance to it and cross-resistance to other anti-cancer therapies [38]. Indeed, 104-day treatment with DAC generates DAC-resistant HCT116 cells. Moreover, the IC50 value of DAC was increased up to 100-fold in DAC-resistant HCT116 cells, and the inhibition of DNA methyltransferase 1 protein level was absent in these cells. CRC patients with high microsatellite stability (MSS) show poor anti-tumor immune response and are characterized by a “cold tumor” microenvironment. Interestingly, the treatment with DAC can drive the 'cold' microenvironment towards a 'hot' immune phenotype by upregulating tumor-associated antigens (TAA) like NY-ESO-1. A combination of DAC with NY-ESO-1-specific TCR-T cells was suggested as an innovative synergistic therapeutic strategy with a significant effect in CRC treatment [39].Therefore, the main aim of our study is to investigate the effect of DAC on immune-related molecules, PD-L1, NY-ESO-1, and on stemness markers in the primary 1076 Col and the metastatic 1872 Col cells to understand its role as a therapeutic agent for CRC.

## Methods

### Cell lines and culture conditions

The primary 1076 Col cell line was established from a poorly differentiated colon adenocarcinoma patient [40]. The metastatic 1872 Col was generated from tumor liver metastases of a colorectal cancer patient [41]. Both cell lines were generously provided by Dr. Cristina Maccalli (Laboratory of Immune and Biological Therapy, Research Branch, Sidra Medicine, Doha, Qatar). Both sequencing (for CANgenes showing differences in the two cell lines) and HLA typing have been performed to verify the identity of both cell lines and confirmed that they are derived from a cancer origin (CANgenes) and from the corresponding patients (HLA typing), respectively [42]. The cell lines were maintained in *in-vitro* cell culture in advanced RPMI 1640 medium (Gibco, 12633-012) supplemented with 10% heat-inactivated FBS (Gibco, 11550356), 1% penicillin/streptomycin (Gibco, 15140-122), and 1% L-glutamine (Gibco, 35050-038). The cells were grown in a humidified incubator at 37 °C and 5% CO_2_.

### In-vitro DAC treatment

Decitabine (DAC) was purchased from Sigma-Aldrich Chemie GmbH (Buchs, Switzerland, A3656). The drug was solubilized with 20 ml of sterile water to obtain a 1 mM stock solution and was kept at 4 °C protected from light until further use. Both cell lines were seeded at a density of 7 × 10^5^ cells in T75 flasks and placed overnight at 37 °C in a 5% CO_2_ incubator. After 24 h, the culture medium was replaced with a fresh medium containing 5 and 10 μM of DAC. The drug was then added every 12 h for 48 h. After this, the cells were maintained in a complete advanced RPMI medium without DAC for 48 h to accumulate the synthesized proteins.

### Proliferation assay by real-time cell analyzer (RTCA)

To observe the growth of CRC cells under different treatment doses in real-time, we first plated the 1076 Col and 1872 Col cells as a monolayer with 5000 and 10,000 cells per well, respectively, as published previously [43], followed by treatment with different doses of DAC (2.5, 5, 10 and 20 µM). The real-time cell analyzer and E-plate 16 (RTCA; xCELLigence, Roche, San Diego, CA, USA) were used to determine the cell index of treated and untreated CRC cultured cells. The cell culture plates used in the RTCA technique are coated with gold microelectrode biosensors that measure electrical impedance when cells adhere to them causing an alternating current. The cell index (CI) reflects the impedance induced by adherent cells. Thereby, CI indicates cell growth. Cell index is calculated as follows:

CI = impedance at time point n (end of the experiment)- impedance in the absence of cells (culture media alone)/ nominal impedance (designed impedance of device).

### Western blot analysis

After DAC treatment, 1076 Col and 1872 Col cells were collected and lysed with RadioImmuno-Precipitation Assay (RIPA) lysis buffer supplemented with 1X protease phosphatase cocktail inhibitors (74106, Roche). Lysates were clarified via centrifugation at 14,000 g at 4 °C for 15 min, and the protein concentrations were measured using the Pierce BCA protein assay (A53226, Thermo Scientific). The total proteins were separated using 4–15% Mini-PROTEAN TGX Gels (4561083, BIO-RAD), transferred onto Trans-Blot transfer membranes (1704156, BIO-RAD), blocked with 1X clear milk (37587, Thermo Scientific), and incubated with specific antibodies: anti-PD-L1 (563738, BD Biosciences), anti-NY-ESO-1 (NB590.04, XBiotech), anti-LC3 A/B (4108 s, Cell Signaling), anti-P62 (5114S, Cell Signaling), anti-Beclin 1(PA1-16857, Invitrogen), anti-caspase-3 (9668 s, Cell Signaling) and anti-cleaved caspase-3 (9664 s, Cell Signaling). The stemness markers CD44 and Nanog (3570 and 4903, Cell signaling) were also evaluated using specific antibodies at 1:1000 dilutions. Anti-β-actin (4970L, Cell Signaling) was used as a housekeeping protein control. After incubation, the membranes were washed and incubated with corresponding secondary antibodies (diluted at 1:2000 dilution) and detected with a Clarity Western ECL Substrate (170–5061, BIO-RAD). The blot was then analyzed by a ChemiDoc™ MP Imaging system (17001402, Bio-Rad Laboratories) and quantified using Image J software (NIH, MD, USA).

### Flow cytometry

In the following experiments, all samples were acquired using BD LSR Fortessa and the data were analyzed using BD FACS DIVA software.

#### Analysis of PD-L1 expression

1076 Col and 1872 Col cells were seeded at a density of 7 × 10^5^ cells in T75 flasks and incubated overnight at 37 °C in a 5% CO_2_ incubator. The treatment with 5 μM DAC was performed as described above in the Methods, section “In-vitro DAC treatment.” At the end of the treatment, the cells were harvested by trypsinization; 0.5 to 1 × 10^6^ cells were washed with PBS (1610780, BIO-RAD) and then centrifuged at 1300 rpm for 10 min. Cell pellets were suspended in PBS (1610780, BIO-RAD) and transferred into FACS tubes. The cells were stained with BV421 mouse anti-Human PD-L1 antibody (563738, Becton–Dickinson) or with the isotype control BV421 mouse IgG1ĸ (562438, Becton–Dickinson), then incubated for 30 min, in the dark, at 4 ˚C. After incubation, the cells were washed twice with cold PBS and analyzed.

#### Analysis of stemness markers expression

1076 Col and 1872 Col cells were seeded at a density of 7 × 10^5^ cells in T75 flasks and incubated overnight at 37 °C in a 5% CO_2_ incubator. The treatment with 5 μM DAC was performed as described above in the Methods, section “In-vitro DAC treatment.” At the end of the treatment the cells were harvested by trypsinization then 3 × 10^6^ cells were washed with PBS (1610780, BIO-RAD), then centrifuged at 1300 rpm for 10 min. Cell pellets were suspended in PBS then transferred into FACS tubes. The cells were stained with antibodies against human CD44 (562890, BD Biosciences, USA), CD133 (130–112-157, Miltenyi Biotec, USA) and Nanog (562259, BD Biosciences, USA) then incubated in the dark, for 30 min at 4 °C. After incubation, the cells were washed twice with cold PBS and analyzed.

#### Analysis of cell autophagy

1872 Col and 1076 Col cells were seeded at a density of 7. 10^4^ cell/ml per well in 6-well plates and were cultured overnight. The cells were then treated with DAC (5 µM) for 48 h. We used MDC labelling (D4008, Sigma Aldrich) for staining of autophagic vacuoles and estimating the number of cells undergoing autophagy. MDC was added to the cells at a final concentration of 50 µM per well during 15 min at 37 °C. At the end of the incubation, the cells were harvested by trypsinization and washed twice with PBS. The cells were then resuspended in 1 ml PBS and counted. 1 × 10^6^ cells were centrifuged at 300 g for 5 min, resuspended in 250–300 µL PBS per FACS tube containing 1 µl of Propidium Iodide (PI) and incubated for 5 min at RT in the dark. We used PI to stain for living cells and gate on this population. After PI staining, the cells were analyzed.

#### Analysis of cell cycle analysis

After treatment with DAC for 48 h, approximately 1 × 10^6^ cells were collected, washed with PBS (1610780, BIO-RAD), fixed, and permeabilized using 70% cold ethanol, then kept overnight at 4 ˚C. Samples were then incubated with propidium iodide (R37169, Thermo Scientific) for 30 min, and the distribution of cells in the different phases of the cell cycle was analyzed.

### qRT-PCR assay

Total RNA was extracted from the 1076 Col and 1872 Col CRC cell lines using RNEasy Mini Kit in accordance with the manufacturer's instructions (74106, Qiagen). Briefly, the concentration of isolated RNA was determined using Nanodrop 2000 (Thermofisher Scientific, USA). 2 μg of extracted RNA was then reverse transcribed using the SuperScript IV First-Strand Synthesis Kit according to the manufacturer's instructions (18091200, Invitrogen). The cDNA was stored at − 20 °C for performing qRT-PCR. qRT-PCR was performed on the Quant Studio 12 K Flex Real-Time PCR System (Thermofisher Scientific, USA). The expression of targeted genes *NY-ESO-1*, *CD133*, *CD44*, *MSI-1*, *Nanog*, *KLF4* and housekeeping gene *GAPDH* was analyzed using TaqMan "assay on-demand" primers (Additional file 1: Table S1). The cycling parameters were denaturation at 95 °C for 10 min, annealing at 95 °C for 15 s (40 cycles), and extension at 60 °C for 1 min. The comparative C_t_ method was applied to calculate the fold-change of RNA expression in comparison to a control sample.

### Immunocytochemistry staining

The 1076 Col and 1872 Col cells were seeded into 4-chamber culture slides (26,500 cells per chamber) (80424, IBIDI) and kept overnight. After 24 h, the cells were washed twice with PBS (1610780, BIO-RAD), fixed with 1X TF fix/perm buffer (51-9008100, BD Pharmingen) for 15 min at room temperature, followed by staining with Alexa-fluor labeled 12D7 conjugated antibody (1:2000) and then incubated overnight at 4°˚C. After incubation, the cells were washed 5 times with perm/wash buffer (51-9008102, BD Pharmingen), stained with 10 µM Hoechst dye, and kept for 15 min at room temperature. The cells were then washed with PBS and examined by A1Rsi Nikon Eclipse Ti confocal microscope. Samples were scanned with X60/1.4 numerical aperture oil immersion objective lens.

### Statistical analysis

The qRT-PCR and western blot data were acquired from the duplicate experiments and presented as mean ± SD. For experimental analysis, paired two-tailed student’s t-test was applied using GraphPad Prism 8. P value < 0.05 was considered statistically significant.

## Results

### Effect of DAC on the proliferation of CRC cells

**Fig. 1:**
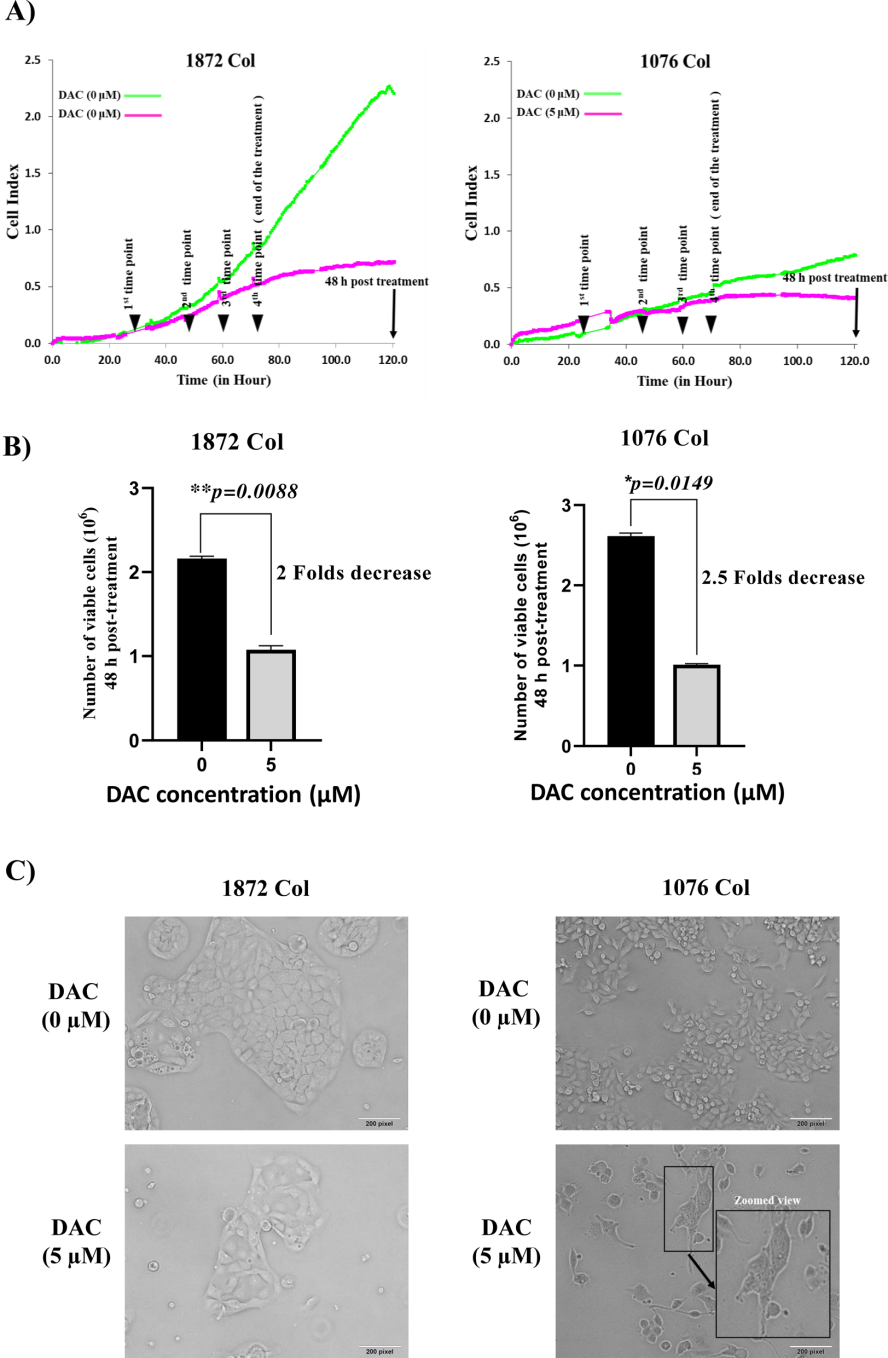
Effect of DAC treatment on cell proliferation and morphology in CRC cell lines, 1076 Col and 1872 Col. **A** Real-time cell proliferation (cell index) analysis of 1076 Col and 1872 Col CRC cells. Cells were grown in a monolayer on top of the electrodes and treated with 5 μΜ of DAC. The real-time cell analyzer was used to determine the cell index, as described in the methods section. **B** Trypan blue exclusion dye assay showed a significant decrease in growth in 1076 Col and 1872 Col CRC cells treated with 5 μΜ of DAC (twofold decrease and 2.5 fold decrease, respectively). **C** DAC treatment resulted in significant morphological changes in both cell lines, such as swelling, stretching, and intracellular particles. Images were captured using a bright-field microscope (Olympus IX51, objective 20×). The data shown are representative of at least three replicate experiments. Scale bar is 200 pixels

The effect of DAC treatment on CRC cell proliferation was assessed using RTCA and trypan blue dye exclusion assays. Both primary 1076 Col and metastatic 1872 Col cells were treated with increasing doses of DAC (2.5, 5, 10, and 20 µM) for 48 h. Based on the RTCA data, the half-maximal inhibitory constant (IC50) value after 48 h of treatment was 4.26 µM and 4.39 for 1076 Col and 1872 Col cells, respectively. Therefore, a concentration of 5 μM of DAC was chosen for further experiments. Our results showed that DAC treatment induced a dose-dependent decrease in cell index (Fig. 1A) and cell number (Fig. 1B) in both cell lines. Treatment with 5 μM DAC decreased cell proliferation in 1076 Col and 1872 Col cells (2.5-fold decrease (*p = 0.0149) and twofold decrease (**p = 0.0088), respectively). In addition, 2.5 μΜ of DAC treatment had no significant effect on cell proliferation, while high doses (10 μΜ and 20 μΜ) were found to be cytotoxic in both cell lines (data not shown). Moreover, compared to untreated cells, treatment with 5 µM of DAC induced morphological changes such as swelling, stretching, and intracellular particles in both cell lines (Fig. 1C).

**Fig. 2:**
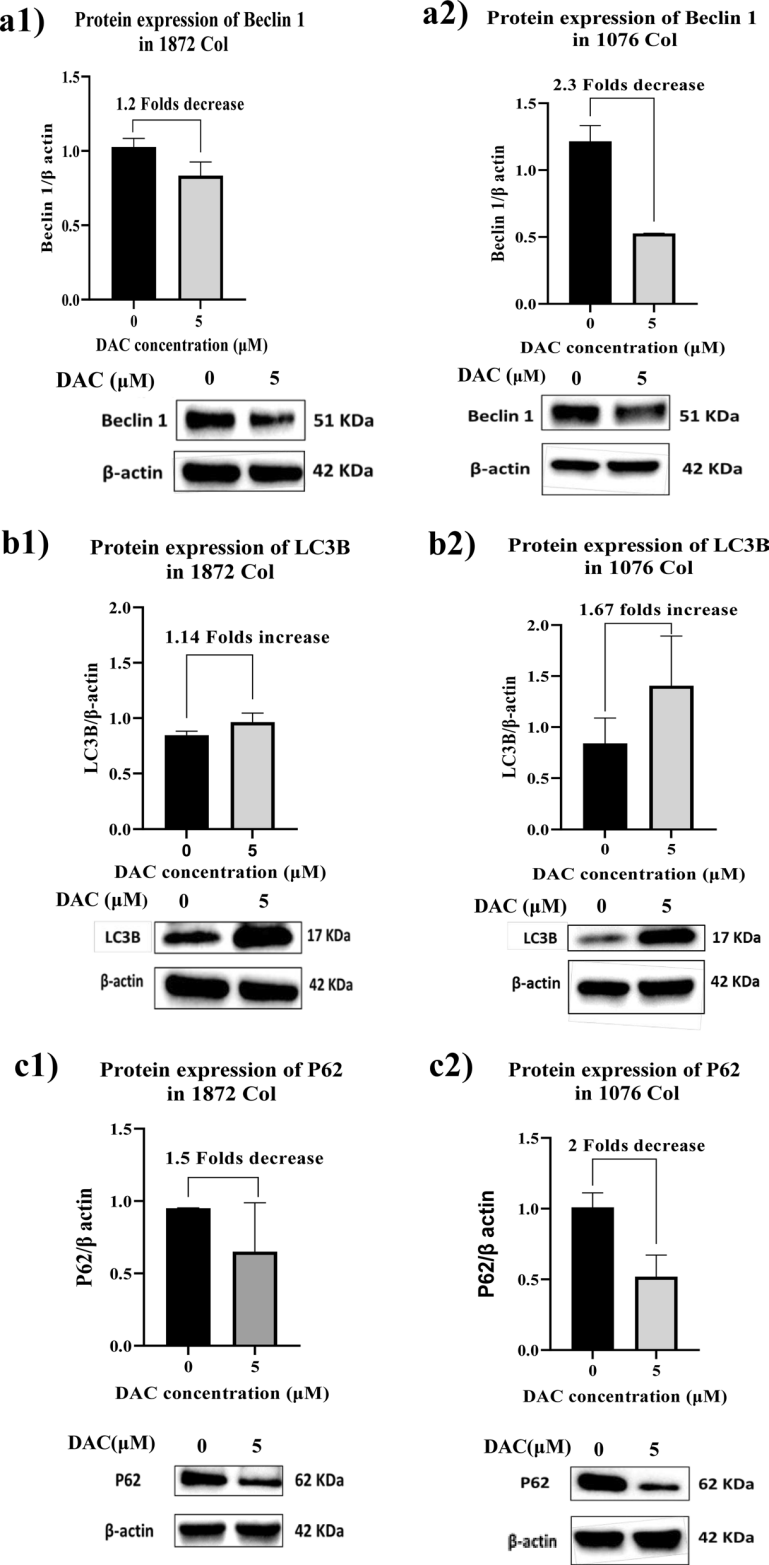
Effect of DAC treatment on autophagy markers. The 1076 Col and 1872 Col cells were treated with DAC (5 μΜ) for 48 h. and then analyzed by immunoblotting. Western blot analysis confirmed induction of autophagy via decreased Beclin-1 level (2.3 fold and 1.2 fold respectively) (**a1**, **a2**), increased LC3B protein level (1.67 fold and 1.14 fold respectively) (**b1**, **b2**), and decreased P62 level after treatment (twofold and 1.5 fold respectively) (**c1**, **c2**). β-actin served as the loading control. The band intensities were quantified using Image J and normalized against β-actin. The data are from duplicate experiments and presented as mean ± SD

To assess the apoptotic markers involved in cell death after DAC treatment, caspase-3 and cleaved caspase-3 were assessed by western blot. The western blot results showed that DAC treatment did not induce caspase 3 expression in 1076 Col cells treated with DAC, but an increase was observed in the 1872 Col cells. The absence of cleaved Caspase 3 in both cell lines, indicates the lack of apoptosis induction (Additional file 2). Our flow cytometry results are in accordance with the above finding (Additional file 3 and Additional file 4). We then investigated the role of autophagy in DAC cytotoxicity in both cell lines. We have demonstrated that Beclin 1 protein, an autophagy marker, was markedly decreased in both 1076 Col (*p = 0.0493) and 1872 Col cells (*p = 0.0393) treated with DAC as compared to untreated cells (Fig. 2a1, a2). To further confirm these results, western blot was performed for the expression of LC3B and P62 proteins (activation markers for autophagy). Treatment with 5 μΜ of DAC induced upregulation of LC3B in 1076 Col (*p = 0.0161) and 1872 Col cells (**p = 0.0016) (Fig. 2b1, b2), and significantly downregulated the expression of the P62 protein in both cell lines; 1076 Col (*p = 0.0498) and 1872 Col cells (**p = 0.0481) (Fig. 2c1, c2). To further confirm the induction of autophagy in DAC-treated 1076 Col and 1872 Col cell lines, we incubated the cells with Mono-dansylcadaverine (MDC) to label autophagic vacuoles in cells. We then stained the cells with PI and analyzed them by flow cytometry. We observed that DAC-treatment induced autophagy in both cell lines, 1076 cell line (0.3% in untreated vs. 4.8% in treated) and in 1872 cell line (0.6% in untreated vs. 4.5% in treated) (Additional file 7).

### Effect of DAC on cell cycle of CRC cells

**Fig. 3:**
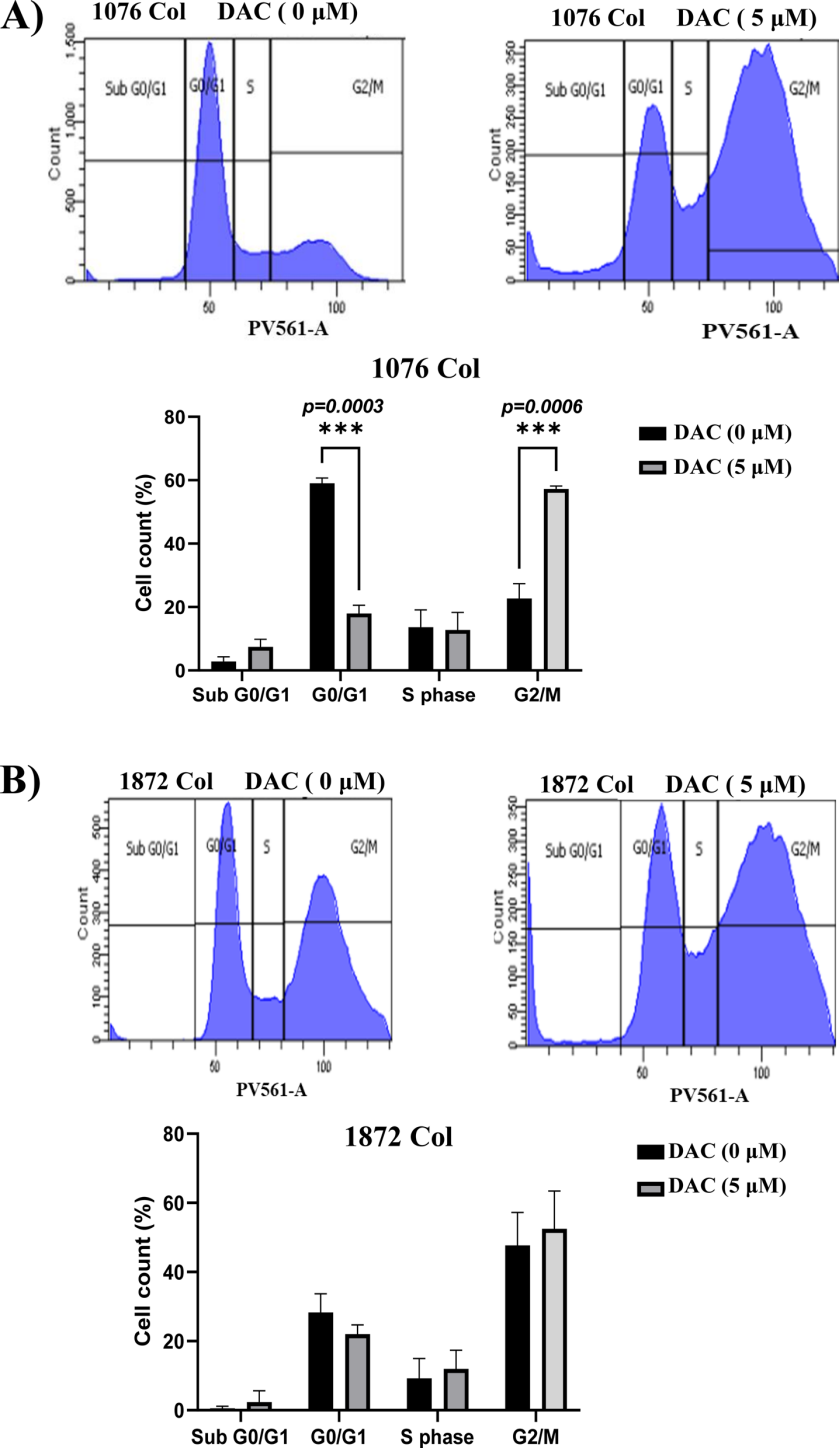
Effect of DAC treatment on 1076 Col and 1872 Col cell cycle. Cell cycle analysis using propidium iodide (PI) staining was performed 48 h. after treatment with 5 μΜ of DAC. Compared to untreated cells, treated cells showed an increased proportion of cells in the G2/M phase and a decrease in G0/G1 phase after DAC treatment in 1076 Col cells (**A**). However, no change was observed in the 1872 Col cells (**B**). The data are from duplicate experiments and presented as mean ± SD

The study of the effect of DAC on cell cycle indicated that treatment with DAC induced a significant increase in the proportion of cells in the G2/M phase and a decrease in the G0/G1 phase in 1076 Col cells (Fig. 3a), while no change was recorded in 1872 Col cell line (Fig. 3b).

### Effect of DAC on stemness markers

**Fig. 4:**
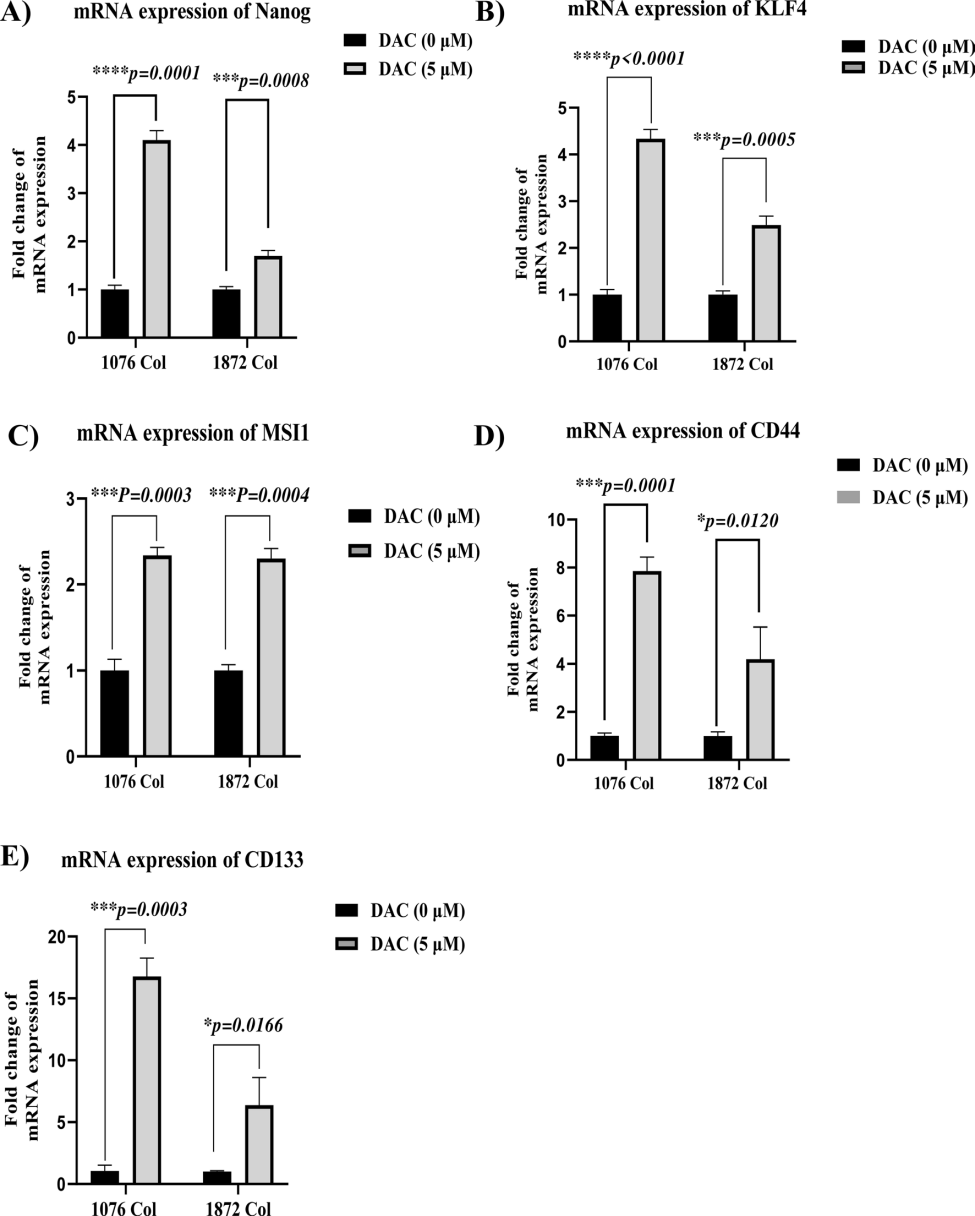
Effect of DAC treatment on the expression of stemness markers in the 1076 Col and 1872 Col CRC cell lines. RT-qPCR showed an up-regulation of mRNA level of Nanog (***p < 0.0001, ***p = 0.0008) (**A**), KLF4 (***p˂0.0001, ***p = 0.0005) (**B**), MSI1 (***p = 0.0003, ***p = 0.0004) (**C**), CD44 (***p = 0.0001, *p = 0.0120) (**D**), and CD133 (***p = 0.0003, *p = 0.0166) (**E**), respectively in both cell lines after DAC treatment. The results are represented as mean + SD from two independent experiments done in duplicates (Paired t-test)

To demonstrate the effect of self-renewal and pluripotency in DAC-treated 1076 Col and 1872 Col cell lines, we investigated the expression of stemness-related transcriptional factors, Nanog, KLF4, and MSI-1 in addition to other stemness-related surface markers (CD44 and CD133). Treatment with DAC significantly increased the mRNA levels of the pluripotency markers, Nanog (Fig. 4A), KLF 4 (Fig. 4B), and MSI-1(Fig. 4C) in 1076 Col and 1872 Col (Fig. 4C) cell lines, respectively. In addition, DAC treatment also significantly upregulated the expression of CD44 (Fig. 4D) and CD133 (Fig. 4E) in 1076 Col and 1872 Col cell lines, respectively. The stemness markers expression was further evaluated at the protein level by flow cytometry and western blot analysis. Our flow cytometry results demonstrated that in 1076 Col cell line, DAC-treatment significantly increased the expression of CD44 (Additional file 8), CD133 (Additional file 8) and Nanog (Additional file 8). DAC treatment did not affect CD44 expression in 1872 cells (Additional file 9). However, the percentage of 1872 Col cells expressing CD133 and Nanog was upregulated after DAC-treatment (67.7% in untreated cells vs. 83.4% after DAC treatment and 0.9% in untreated cells vs. 25.9% after DAC treatment respectively) (Additional file 8). Our western blot results revealed that CD44, Nanog and KLF4 were upregulated in both cell lines (1872 and 1076), treated with DAC (Additional file 9).

### Effect of DAC on the expression of the immune checkpoint PD-L1 molecule

**Fig. 5:**
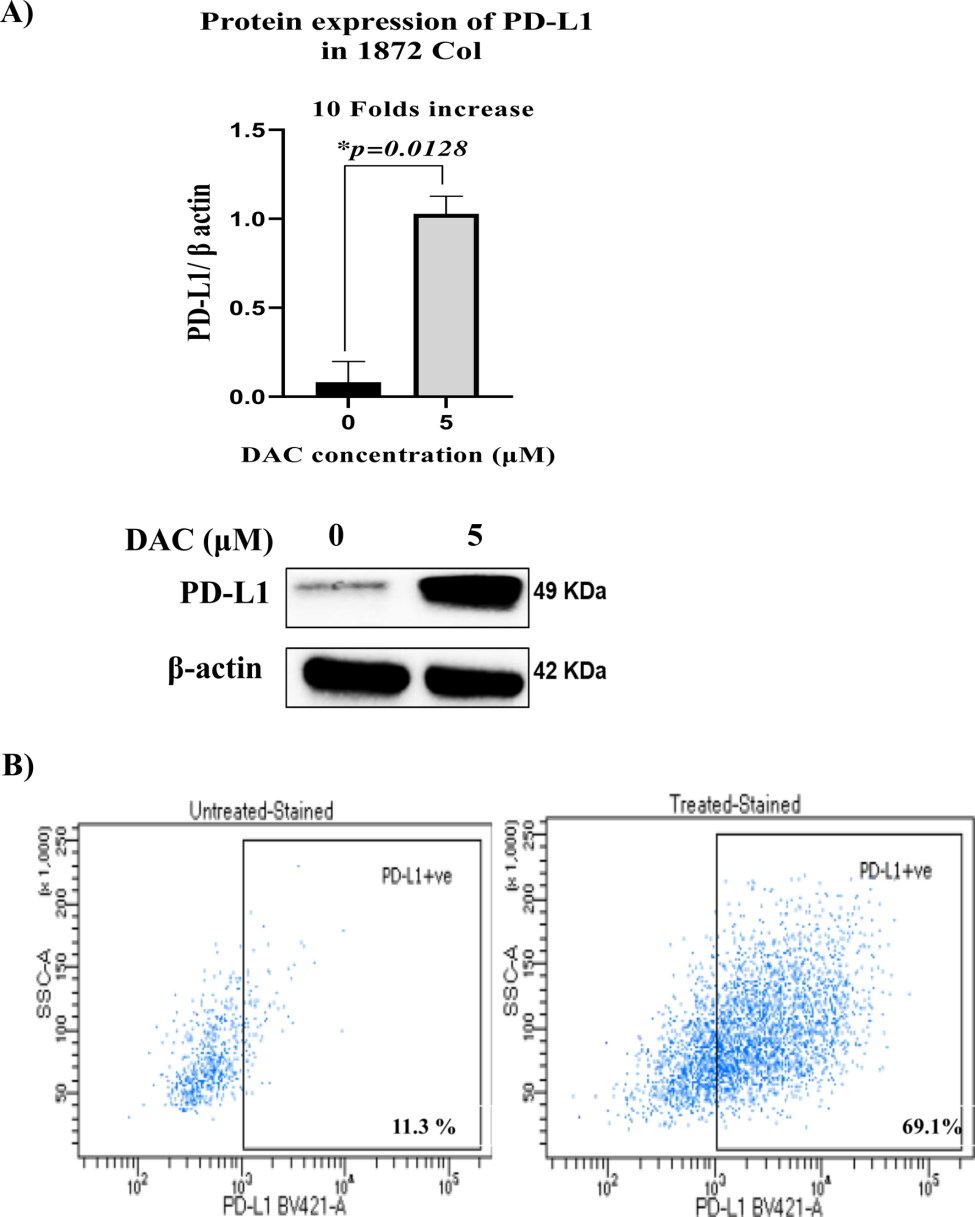
Effect of DAC treatment on PD-L1 expression in the 1872 Col. DAC increased PD-L1 expression in the 1872 Col as shown by Western blot (tenfold increase; *p = 0.0128) (**A**) and Flow cytometry (11.3% in untreated cells vs. 69.1% after DAC treatment) (**B**). The data are from duplicate experiments and presented as mean ± SD (Paired t-test)

We evaluated the effect of DAC on the expression of the immunoinhibitory checkpoint PD-L1 marker in the 1076 Col and 1872 Col cell lines. Protein level expression of PD-L1 was evaluated by western blot and flow cytometry assays. DAC treatment markedly upregulated the expression of PD-L1 in the 1872 Col cell line at the total protein level (Fig. 5A) and at the surface expression level (Fig. 5B). However, PD-L1 had no marked or significant induction/upregulation in the 1076 Col cells (Data not shown).

### Effect of DAC on the expression of the immunogenic NY-ESO-1 cancer-testis antigen

**Fig. 6:**
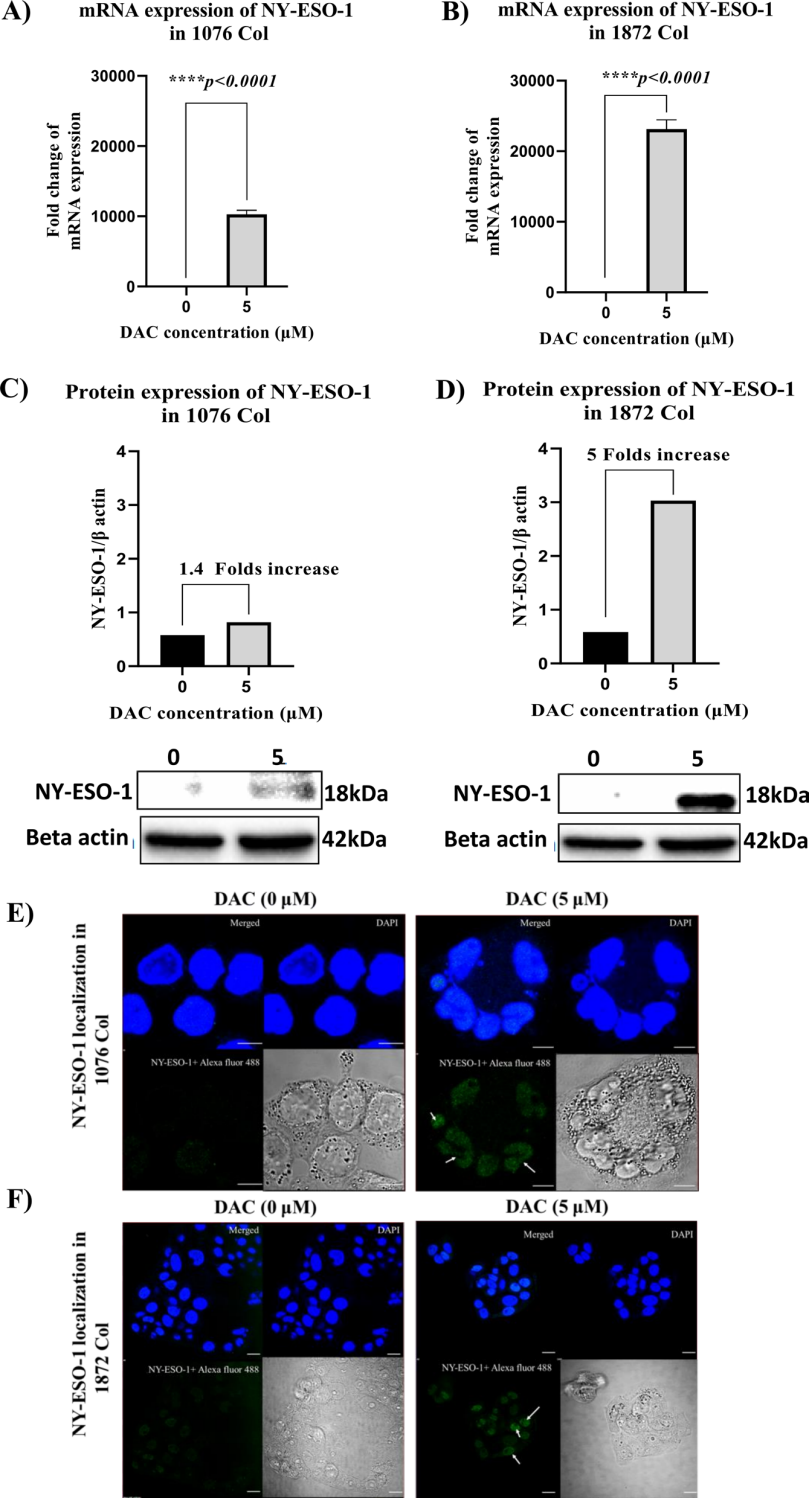
Effect of DAC treatment on the expression of the NY-ESO-1 tumor antigen in CRC 1076 Col and 1872 Col cell lines. (**A**, **B**) RT-qPCR analysis showed an induction of NY-ESO-1 in both 1076 Col and 1872 Col after 5 μΜ of DAC treatment. (**C**, **D**) Western blot analysis showed a significant induction of NY-ESO-1 in 1872 (fivefold, ****p = 0.0001) than in 1076 (1.4-fold, ****p = 0.0001) Col following treatment with DAC. (**E**, **F**) Confocal microscopy images showing the localization of NY-ESO-1 (white arrows) in 1076 Col and 1872 Col cells treated with 5 μM of DAC compared to the untreated cells. Cells were treated with DAC for 48 h. and then stained with Alexa-fluor labeled 12D7-conjugated antibody as described in the methods section. Fluorescence images were captured at 60X magnification. Arrows presented in the confocal images indicate that induced NY-ESO-1 is located at the nucleus. The data are from duplicate experiments and presented as mean ± SD. Scale bar is 10 μm in (**E**). Scale bar is 20 μm

After DAC treatment, the expression of NY-ESO-1 mRNA was increased by ~ 10,000 fold and ~ 23,000 fold in both 1076 Col and 1872 Col cell lines, respectively (Fig. 6A, B). Furthermore, western blot analysis showed that DAC treatment induced a fivefold increase in the NY-ESO-1 protein expression in the 1872 Col cells (Fig. 6D). However, NY-ESO-1 expression was minimum (1.4-fold) in the 1076 Col cells (Fig. 6C). Cellular localization of NY-ESO-1 protein by immunocytochemistry showed specific binding of the anti-NY-ESO-1 antibody to intracellular NY-ESO-1 protein in treated primary 1076 Col (Fig. 6E) and metastatic 1872 Col (Fig. 6F) cell lines. The intensity of the expression of NY-ESO-1 protein after DAC treatment was confirmed in both cell lines (Additional file 5 and Additional file 6). Interestingly the accumulation of the NY-ESO-1 protein was mainly observed inside the nucleus of the treated cancer cells.

## Discussion

DAC is a DNA demethylating agent that activates tumor suppressor genes silenced by aberrant promoter methylation. DAC has been clinically effective in treating several hematological malignancies, such as MDS and AML [33–36]. The therapeutic potential of DAC for treating various types of solid tumors, as monotherapy or in combination with other therapeutic strategies, is under investigation [37]. In the present study, we investigated the effect of DAC on one primary (1076 Col) and one metastatic (1872 Col) colorectal cancer cell lines isolated and established *in-vitro* from patients' tumor tissues. Our results showed that DAC inhibited the growth of both cell lines by 2.5 and twofold, respectively. Several studies have reported various mechanisms associated with DAC cytotoxicity on tumor cells, including the reactivation of tumor suppressor pathways [44, 45], such as apoptosis and autophagy. In our study, apoptosis did not mediate the cytotoxicity of the cell lines. However, the autophagy markers Beclin 1, LC3B, and P62 were found to be modulated by DAC treatment. *Beclin 1* is the first identified ATG gene in mammalian cells and the more commonly investigated gene involved in autophagy. Several studies on ovarian, breast, and prostate cancers have reported the downregulation of Beclin 1 as a mechanism of tumor cell proliferation [46–48]. The role of Beclin 1 in CRC tumor progression is unclear. A study by Ahn et al. revealed that in tumor tissues from 103 CRC and 60 gastric cancer patients, the expression of Beclin 1 was found to be 95% and 83%, respectively. In contrast, minimal /no expression of Beclin 1 was observed in healthy gastric and colorectal tissues [49]. In our study, Beclin 1 was markedly decreased in both primary and metastatic cell lines compared to untreated cells. Two autophagy markers, LC3B and P62, were investigated further to decipher the role of autophagy in DAC treatment. Studies have shown that the LC3 protein leaves the cytoplasm to the autophagosomal membranes during autophagy. After that, the autophagosome joins the lysosome to create autolysosome, where the autophagosome vesicle and its contents are broken down. On the other hand, the P62 protein interacts with ubiquitinated proteins leading to continuous damage of LC3B. Thus, reduced levels of P62 are consequently linked to an active autophagy process. We have found that DAC treatment decreased the expression of Beclin 1 and P62 while it increased the expression of LC3B, indicating the activation of autophagy (Fig. 2). These results are in agreement with previous investigations in pancreatic [50] and breast cancers [51]. On the other hand, studies in lung cancer, human synovial sarcoma cells, and leukemia cells have shown that overexpression of Beclin 1 promotes cell death [52–54]. Thus, the role of Beclin 1 expression on cell viability varies according to cancer cells with different histological origins. Further studies are warranted to understand the role of Beclin 1 in DAC treatment.

We further investigated the effect of DAC on cell cycle. We have shown that DAC induced an accumulation of cells in the G2/M phase in the primary 1076 Col cells but not in the metastatic 1872 Col cells. This result is in agreement with previous reports, which showed that DAC-induced cell cycle accumulation in primary myeloma, bladder transitional cell carcinoma, and glioma cells [55–57]. The failure of the induction of an accumulation in the G2/M phase by DAC in the metastatic 1872 Col cells may be due to the gain of protection of cellular adaptations and mutations by the metastatic cancer cells, which may be playing a role in the blocking of cell cycle arrest. However, further studies on other metastatic cell lines are needed to validate this observation.

Chemotherapeutic drugs are well known to affect the stemness characteristics of tumor cells, thus facilitating the self-renewal and differentiation potential of the cells into heterogeneous lineages [58]. These lineages have been evidenced to induce chemotherapy and radiotherapy resistance in cancer cells leading to relapse and treatment failure [59–61]. Several stemness markers have been utilized to identify cancer stem cells. Among these, the transmembrane glycoprotein CD133 and the cell-surface glycoprotein CD44 have been demonstrated to be associated with cell migration, invasion, metastasis, and therapeutic resistance [62–65]. Studies have shown that higher expression of CD133 in CRC is predictive of poor response and also, resistance to chemotherapy and radiation therapy [15, 66, 67]. Furthermore, a study by Dallas et al. has demonstrated that cells with high CD44 and CD133 expression exhibited stem cell-like features and resistance to 5-FU treatment [68]. In our study, the treatment with DAC induced the up-regulation of the expression of CD44 and CD133 in the primary 1076 Col cells (7.85 fold and 16.77 fold, respectively). However, only a 4.19 fold (CD44) and 6.36 fold (CD133) increase was observed in the metastatic 1872 Col cells, indicating the increased upregulation of stemness markers in primary cells, probably to provide the cells with proliferation and survival properties. Another stemness marker is the transcription factor KLF4, a gene that reprograms pluripotent stem cells. In cancer, KLF4 can have a dual role according to the type of cancer or tumor stage, acting either as a tumor suppressor or oncogene [69]. Recent studies have demonstrated that KLF4 inhibits CRC proliferation [70] and sensitizes cells to chemotherapy by chemotherapy-mediated G2/M cell cycle arrest [33]. Interestingly, we have demonstrated a higher upregulation of KLF4 in the primary 1076 Col (4.33 fold) with an accumulation of cells in the G2/M phase. Another key transcription factor known as Nanog has been demonstrated to be involved in self-renewal and pluripotency in CRC cells [71]. Indeed, Nanog overexpression has been linked to colony formation, a poor prognosis development of stem cell properties in CRC [71]. Moreover, Nanog inhibition has been associated with increased sensitivity to the 5-FU drug in CRC cell lines, indicating its importance in targeted therapeutics [71]. We found that DAC treatment increased the expression of the gene encoding for Nanog by twofold higher in the primary 1076 Col cells than in the metastatic1872 Col cells. These results indicate the efficacy of DAC in inducing markers that can help to sensitize the primary CRC cells to chemotherapy. Another stemness marker known as Musashi-1 (MSI-1) was also analyzed in our study. MSI-1 is documented as a critical oncoprotein in CRC [72]. Studies have shown that knocking down this gene leads to the inhibition of CRC cell proliferation, indicating a possible role of MSI-1 in CRC tumorigenesis [73, 74]. Chiou et al. demonstrated that MSI-1 expression promoted the development of CD44 + /CSCs in CRC and simultaneously increased chemoresistance in the CRC cells through the inhibition of anti-apoptotic effect via the formation of stress granules (SG), facilitating stress resistance activities of the cells [75]. Our results agree with the findings of this study, where DAC treatment increased the expression of MSI-1 in both primary and metastatic CRC cells by 2.3 fold but did not induce any apoptotic effect in these cells. Therefore, MSI-1 can be considered a potential therapeutic target in CRC.

Our study also aimed to decipher the immunomodulatory pathways affected by DAC and their role in cancer therapeutics. PD-L1 is a key immunoregulatory protein expressed on tumor cells. When PD-L1 interacts with its receptor PD-1, it suppresses CD8 + cytotoxic T cells necessary for tumor killing [76]. Immunotherapies targeting PD-1 and its ligand PD-L1 have shown promising clinical results in various cancers, including non-small cell lung cancer and bladder cancer [77, 78]. However, most CRC patients, particularly those with microsatellite stable (MSS) tumors, do not respond to an immune checkpoint inhibitor [79, 80]. Recent investigations have demonstrated that the therapeutic efficacy of ICI is superior in patients with substantial intratumoral CD8 + T cell infiltration, tumor mutational load, and the expression of PD-L1 by tumor cells [81–84]. Huang et al. reported that CRC patients with higher PD-L1 expression showed improved survival, and this was positively correlated with intertumoral CD8 + T cell infiltration [85]. Therefore, developing strategies to enhance tumor PD-L1 expression would improve the clinical response in cancer patients. In line with previous findings [86], our study indicates that DAC treatment significantly upregulated the expression of PD-L1 in the metastatic 1872 Col cells, while no changes were recorded in its expression level in the primary 1076 Col cell line. This upregulation of PD-L1 in response to DAC has been reported as a mechanism of adaptive immune resistance that usually emerges in CRC as a result of exposure to chemotherapeutic drugs such as FOLFOX [87, 88]. Our findings highlight the ability of metastatic CRC cells to evade chemotherapy via the upregulation of PD-L1. This interesting finding sheds light on the importance of using DAC to upregulate tumor expression of PD-L1, which PD-1/PD-L1 inhibitors can easily target.

We further investigated the role of DAC in enhancing/inducing the expression of the NY-ESO-1 cancer-testis antigen. NY-ESO-1 is the most immunogenic and well-studied tumor antigen, and its expression in cancers elicits a robust anti-tumor response [89]. NY-ESO-1 expression is widely and variably distributed among various tumor types but has been reported to be poorly expressed in CRC [29]. We have demonstrated that DAC treatment induced high expression of the NY-ESO-1 antigen in the metastatic 1872 Col cells with less upregulation/induction in the primary 1076 Col cells (Fig. 6). Consistent with our results, Coral et al. showed that DAC treatment induced the expression of NY-ESO-1 in renal cell carcinoma, and this effect persisted up to 60 days after the treatment [90].

## Conclusions

In conclusion, our results show that the poorly immunogenic environment in metastatic CRC can be enhanced by induction of NY-ESO-1 antigen and upregulation of PD-L1 expression. These findings are extremely promising, and they form primary evidence for suggesting a novel combination therapy using DAC therapy with immunotherapy to enhance the clinical outcome in CRC patients. Along this line, we have recently shown that the immunological monitoring of NY-ESO-1-specific T-cell response is a potential biomarker of clinical response to the anti-PD-1 treatment [91–93]. We observed that NY-ESO-1-specific T cells response was increased at stable disease stage but significantly decreased at progression in a patient with head and neck Squamous cell carcinoma treated with Nivolumab [91]. We have also demonstrated that NY-ESO-1-specific T cells were significantly upregulated in a patient with metastatic gastric cancer with complete response after a treatment combining radiation therapy with the anti-PD-L1 mAb pembrolizumab [92].

## Supplementary Information

**Additional file 1: Table S1.** Commercially available TaqMan primers are used for reverse transcription-quantitative polymerase chain (RT-qPCR). The primer details are included in the below table.

**Additional file 2: Supplement 1.** Effect of DAC treatment on apoptosis markers. Western blot analysis showed no changes in caspase 3 and cleaved caspase in 1872 Col (M) (A) and 1076 Col (P) (B) cells after DAC treatment.

**Additional file 3: Supplement 2.** Effect of DAC treatment on apoptosis in 1872 Col (M) cells. Untreated and DAC treated cells were stained with Annexin/PI and apoptosis was checked using flow cytometry analysis. Our results showed no apoptosis after treatment of 1872 Col (M) cells with DAC.

**Additional file 4: Supplement 3.** Effect of DAC treatment on apoptosis in 1076 Col cells. Untreated and DAC treated cells were stained with Annexin/PI and apoptosis was checked using flow cytometry analysis. Our results showed no apoptosis after treatment of 1076 Col cells with DAC.

**Additional file 5: Supplement 4.** Effect of DAC treatment on the expression of NY-ESO-1 in 1076 Col. The fluorescence intensity profiles (al: before treatment, bl: after DAC treatment) as well as surface intensity (a2: before treatment, b2:after DAC treatment) derived from their corresponding fluorescence pictures in figure 6 E, confirm the induction of NY-ESO-1 after DAC treatment.

**Additional file 6: Supplement 5.** Effect of DAC treatment on the expression of NY-ESO-1 in 1872 Col. The fluorescence intensity profiles (al: before treatment, bl: after DAC treatment) as well as surface intensity (a2: before treatment, b2: after DAC treatment) derived from their corresponding fluorescence pictures in figure 6 F , confirm the induction of NY-ESO-1 after DAC treatment.

**Additional file 7: Supplement 6.** DAC-induced autophagy in 1872 Col and 1076 Col cells. Cells treated with DAC (5 μM) were stained with MDC and PI and analyzed by flow cytometry for PI negative (live cells) and MDC positive (autophagic cells). Flow cytometry analysis showed that DAC treatment induced autophagy in 1076 Col (0.3% in untreated vs. 4.8% in treated) (A) and 1872 Col (0.6% in untreated vs. 4.5% in treated) (B).

**Additional file 8: Supplement 7.** Effect of DAC treatment on the expression of CD133, CD44 and Nanog in 1872 cells. Flow cytometry analysis showed that DAC treatment did not affect CD44 expression in 1872 cells (A). However, DAC treatment upregulated the expression of CD133 (67.7% in untreated cells vs. 83.4% after DAC treatment) (B) and Nanog (0.9% in untreated cells vs. 25.9 % after DAC treatment) (C). The samples were acquired using BD LSR Fortessa and the data were analyzed using BD FACS DIVA software.

**Additional file 9: Supplement 8.** Effect of DAC treatment on the expression of CD133, CD44 and Nanog in 1076 cells. Flow cytometry analysis showed that DAC treatment significantly increased the expression of CD44 (0.5% in untreated cells vs. 31.4% after DAC treatment) (A), CD133 (2.3% in untreated cells vs. 77.8% after DAC treatment) (B) and Nanog (0.5% in untreated cells vs. 44.3 % after DAC treatment) (C) in 1076 cells. The samples were acquired using BD LSR Fortessa and the data were analyzed using BD FACS DIVA software. (D) Western blot analysis showed that DAC treatment increased the expression of CD44, Nanog and KLF4 in both cell lines tested.

## Data Availability

All datasets used and/or analyzed during experiments are available from the corresponding author on reasonable request.

## Abbreviations

CRC: Colorectal cancer
NY-ESO-1: New York Esophageal Squamous Cell Carcinoma 1
DAC: Decitabine
CSCs: Cancer stem cells
ICI: Immune checkpoint inhibitors
PD-1: Programmed cell death protein 1
PD-L1: Programmed cell death ligand 1
5-FU: 5-Fluorouracil
IRI: Irinotecan
OX: Oxaliplatin
