# Supplementary material for: Treatment with decitabine induces the expression of stemness markers, PD-L1 and NY-ESO-1 in colorectal cancer: potential for combined chemoimmunotherapy
Source: J Transl Med. 2023 Mar 31;21:235. doi: 10.1186/s12967-023-04073-y (PMC10067322; doi:10.1186/s12967-023-04073-y)
Supplement: Supplementary file 1 — Additional file 1: Table S1. Commercially available TaqMan primers are used for reverse transcription-quantitative polymerase chain (RT-qPCR). The primer details are included in the below table. [file 12967_2023_4073_MOESM1_ESM.docx]

**Additional file 1: Table S****1.** Commercially available TaqMan primers are used for reverse transcription-quantitative polymerase chain (RT-qPCR). The primer details are included in the below table.

| **Gene Name** | **Accession number** |
| --- | --- |
| *NY-ESO-1* | HS00265824 |
| *CD133* | HS01009259 |
| *CD44* | HS01045894 |
| *MSI-1* | HS01045894 |
| *Nanog* | HS02387400 |
| *KLF4* | HS00358836 |
| *GAPDH* | HS02786624 |
