# Supplementary figures and images for: Treatment with decitabine induces the expression of stemness markers, PD-L1 and NY-ESO-1 in colorectal cancer: potential for combined chemoimmunotherapy
Source: J Transl Med. 2023 Mar 31;21:235. doi: 10.1186/s12967-023-04073-y (PMC10067322; doi:10.1186/s12967-023-04073-y)

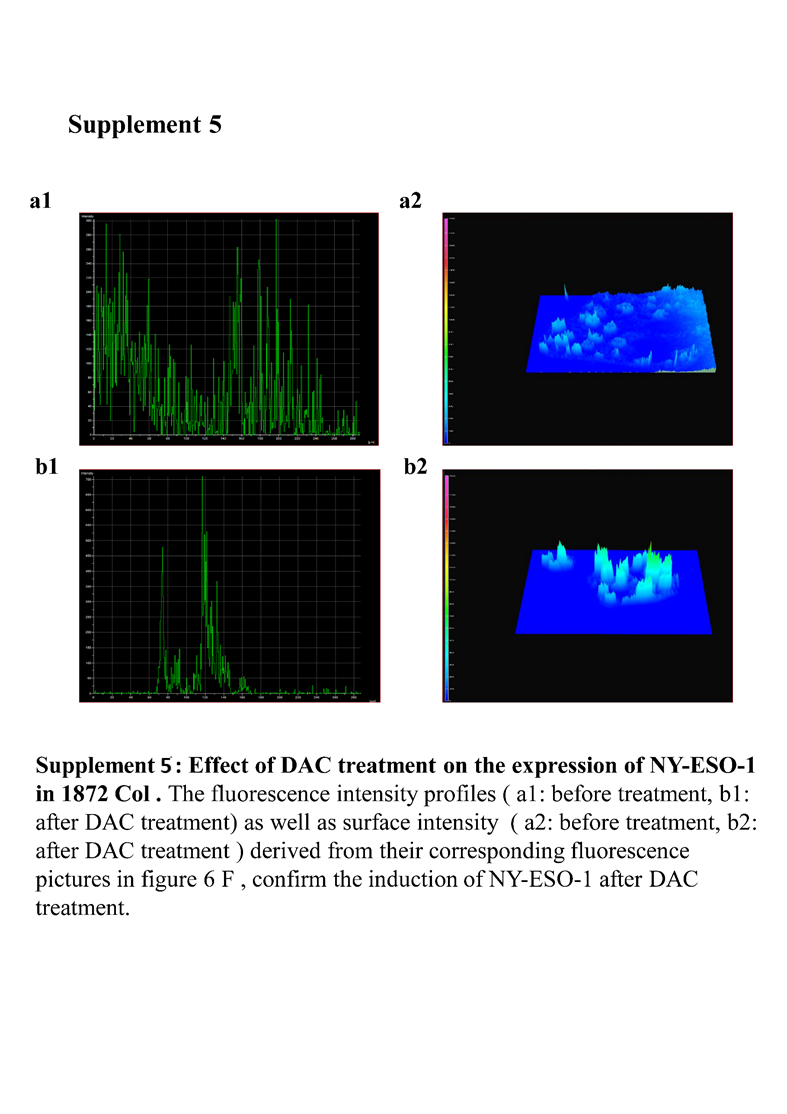

Supplement: Supplementary file 5 — Additional file 5: Supplement 4. Effect of DAC treatment on the expression of NY-ESO-1 in 1076 Col. The fluorescence intensity profiles (al: before treatment, bl: after DAC treatment) as well as surface intensity (a2: before treatment, b2:after DAC treatment) derived from their corresponding fluorescence pictures in figure 6 E, confirm the induction of NY-ESO-1 after DAC treatment. [file 12967_2023_4073_MOESM5_ESM.tif]

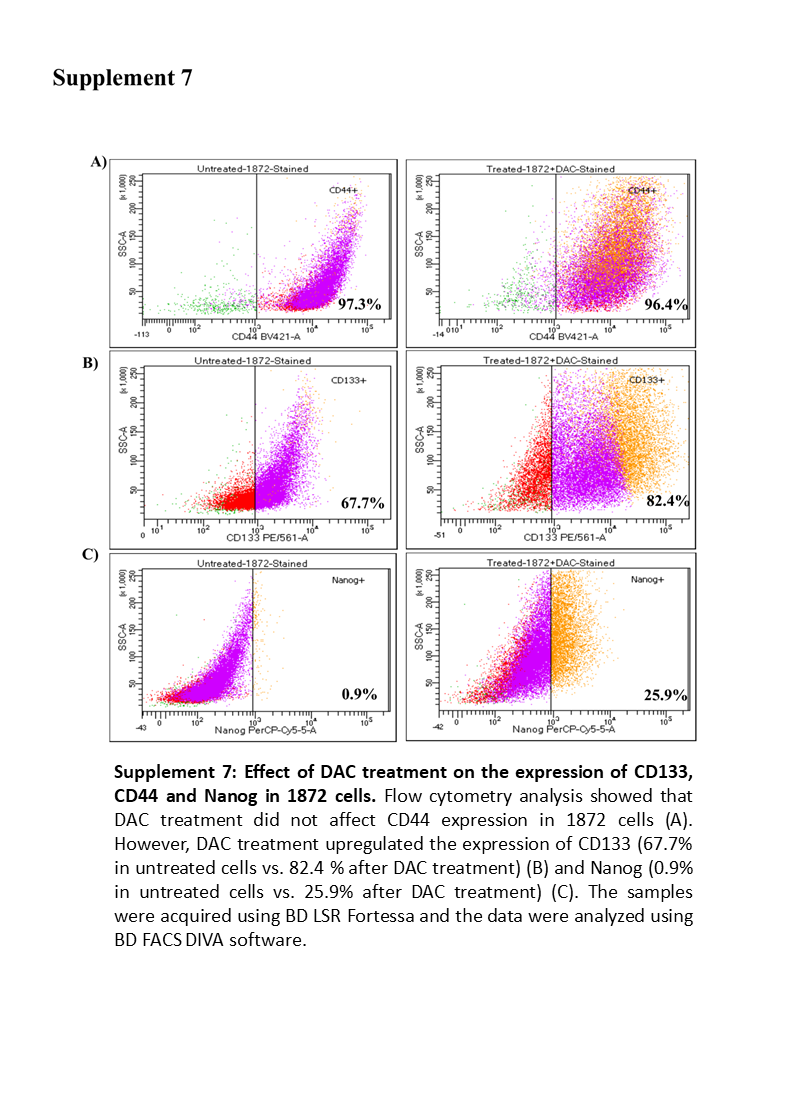

Supplement: Supplementary file 7 — Additional file 7: Supplement 6. DAC-induced autophagy in 1872 Col and 1076 Col cells. Cells treated with DAC (5 μM) were stained with MDC and PI and analyzed by flow cytometry for PI negative (live cells) and MDC positive (autophagic cells). Flow cytometry analysis showed that DAC treatment induced autophagy in 1076 Col (0.3% in untreated vs. 4.8% in treated) (A) and 1872 Col (0.6% in untreated vs. 4.5% in treated) (B). [file 12967_2023_4073_MOESM7_ESM.tif]

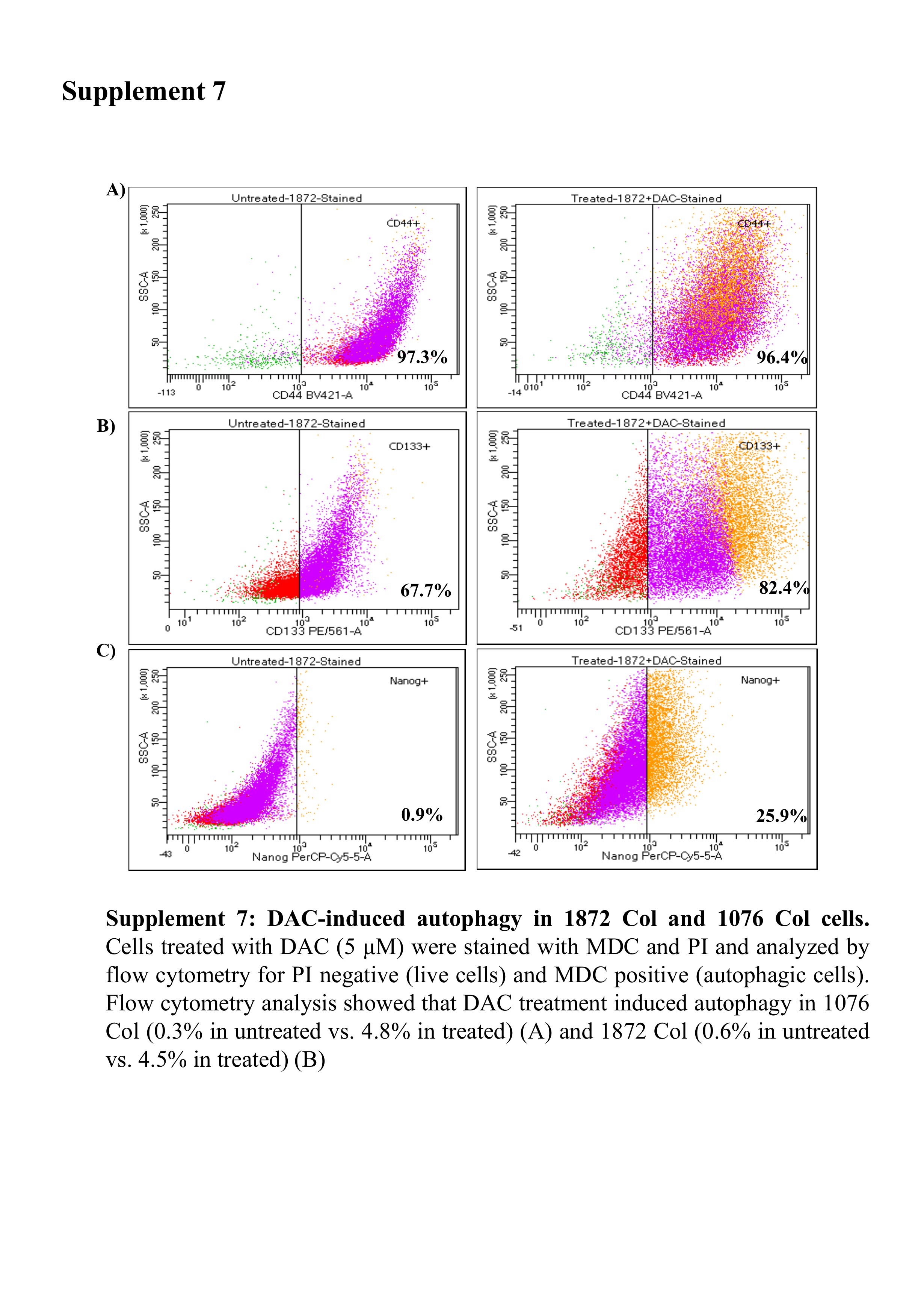

Supplement: Supplementary file 8 — Additional file 8: Supplement 7. Effect of DAC treatment on the expression of CD133, CD44 and Nanog in 1872 cells. Flow cytometry analysis showed that DAC treatment did not affect CD44 expression in 1872 cells (A). However, DAC treatment upregulated the expression of CD133 (67.7% in untreated cells vs. 83.4% after DAC treatment) (B) and Nanog (0.9% in untreated cells vs. 25.9 % after DAC treatment) (C). The samples were acquired using BD LSR Fortessa and the data were analyzed using BD FACS DIVA software. [file 12967_2023_4073_MOESM8_ESM.tif]

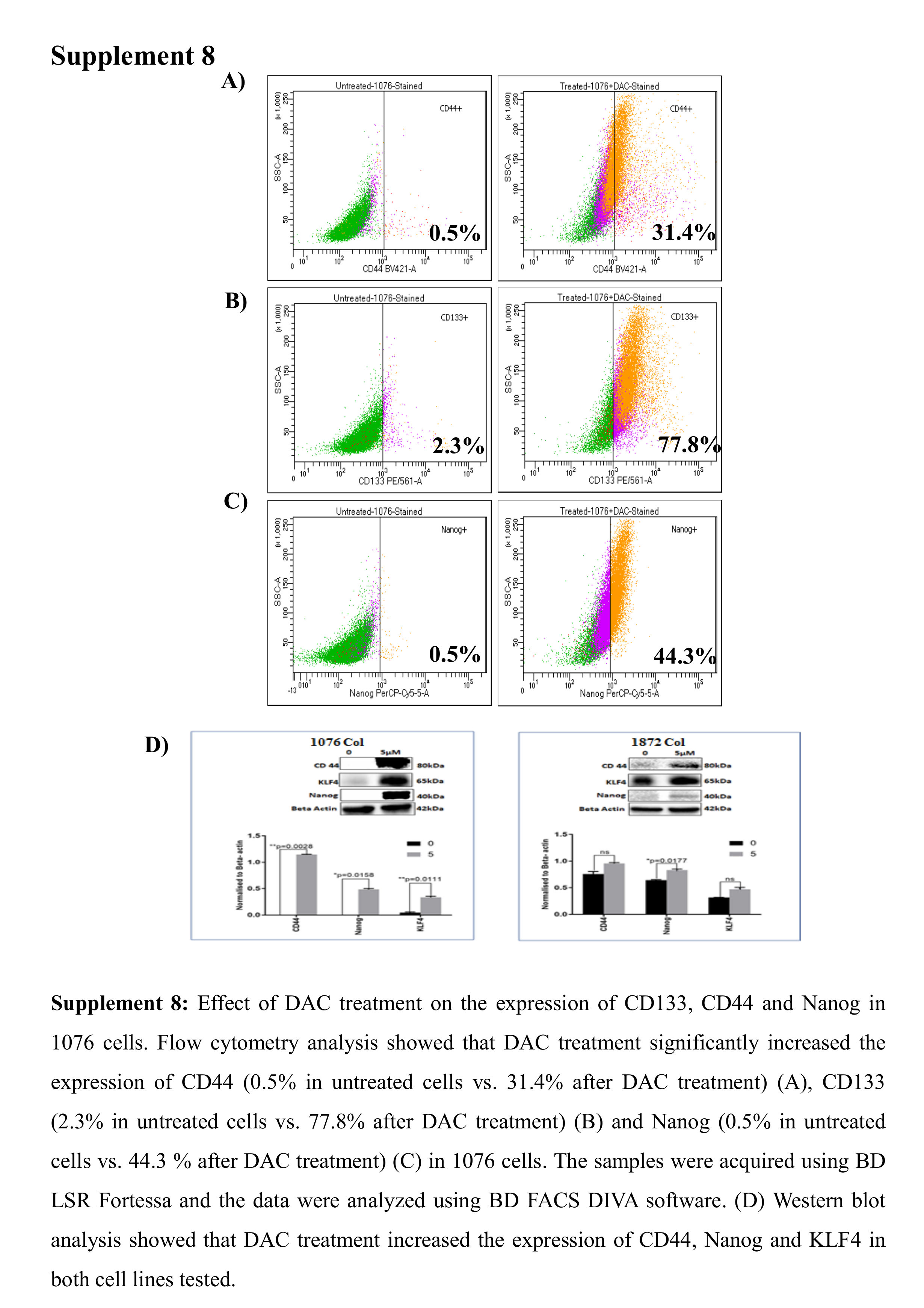

Supplement: Supplementary file 9 — Additional file 9: Supplement 8. Effect of DAC treatment on the expression of CD133, CD44 and Nanog in 1076 cells. Flow cytometry analysis showed that DAC treatment significantly increased the expression of CD44 (0.5% in untreated cells vs. 31.4% after DAC treatment) (A), CD133 (2.3% in untreated cells vs. 77.8% after DAC treatment) (B) and Nanog (0.5% in untreated cells vs. 44.3 % after DAC treatment) (C) in 1076 cells. The samples were acquired using BD LSR Fortessa and the data were analyzed using BD FACS DIVA software. (D) Western blot analysis showed that DAC treatment increased the expression of CD44, Nanog and KLF4 in both cell lines tested. [file 12967_2023_4073_MOESM9_ESM.tif]
